# ﻿A species-group key and notes on phylogeny and character evolution in New Guinean *Exocelina* Broun, 1886 diving beetles (Coleoptera, Dytiscidae, Copelatinae)

**DOI:** 10.3897/zookeys.1131.94205

**Published:** 2022-11-22

**Authors:** Helena Shaverdo, Michael Balke

**Affiliations:** 1 Naturhistorisches Museum, Burgring 7, A-1010 Vienna, Austria Naturhistorisches Museum Vienna Austria; 2 SNSB-Zoologische Staatssammlung München, Münchhausenstraße 21, D-81247 Munich, Germany SNSB-Zoologische Staatssammlung München Munich Germany; 3 GeoBioCenter, Ludwig-Maximilians-University, Munich, Germany Ludwig-Maximilians-University Munich Germany

**Keywords:** Morphology, New Guinea, phylogeny, water beetles

## Abstract

Detailed information about the known species groups of *Exocelina* Broun, 1886 from New Guinea is presented, including species numbers, distribution, and references of species-group diagnoses, keys to the species, and species descriptions. An identification key to all species groups is provided. Phylogeny and morphological character evolution are discussed.

## ﻿Introduction

*Exocelina* Broun, 1886 is a highly diverse genus of diving beetles. Most species occur in running-water habitats, especially low-order streams and habitats associated with wider mountain streams, throughout the Australian, Pacific and Oriental regions. Mainly lentic lifestyles also occur in four independent and not particularly species rich clades (Toussaint et al. 2015).

The genus was proposed by Broun (1886) for his new, most likely epigean species, *Exocelinaadvena*, described from Mokohinau Islands, New Zealand. Later, Broun (1893) recognised it as *Copelatus* and renamed it *C.sharpi* due to homonymy with the Neotropical *C.advena* Sharp, 1882. However, this name also turned to be a junior homonym of another Neotropical species (Branden 1884) and was synonymised with *C.australis* (Clark, 1863) by Zimmermann (1920).

*Exocelina* was infused with new taxonomic life under the name Copelatus (Papuadytes) Balke, 1998. *Papuadytes* was erected based on morphological characters for 31 New Guinean species, with subsequent addition of a Chinese species (Balke and Bergsten 2003) and seven additional New Guinean species (Balke 1999; Shaverdo et al. 2005). The monophyly and generic status of the group were supported following analyses of copelatine phylogeny based on DNA sequence data (Balke et al. 2004, 2007; Bilton et al. 2015). Transferring more and more Australian *Copelatus* species to *Papuadytes* (Nilsson & Fery, 2006) led to the inclusion of *C.australis* (= *Exocelinaadvena*) in *Papuadytes*, and the latter name was recognised as being a synonym of *Exocelina* (see Nilsson 2007). Further investigation showed that the genus has a wide distribution in Australasia: 37 species (including 27 new ones) were recorded from New Caledonia (Wewalka et al. 2010; Balke et al. 2014); one species, *E.cheesmanae* (J. Balfour-Browne, 1939), from Vanuatu (Bilton et al. 2015); one species, *E.parvula* (Boisduval, 1835), from Hawaii (Nilsson 2007); two new interstitial species from Australia (Watts and Humphreys 2009; Watts et al. 2016); one new subterranean species, *E.sugayai*, from Malaysia (Balke and Ribera 2020).

However, New Guinea is the core of species diversity of the genus and, therefore, was the focus of our taxonomic project started in 2012. Since the publication of Shaverdo et al. (2005) on *Exocelina* of the island, 116 new species have been described (Table 1), increasing the number of *Exocelina* in New Guinea to 152 species and the number of *Exocelina* worldwide to 209 species (Nilsson and Hájek 2022). We believe that further extensive fieldwork in New Guinea and careful taxonomic investigation of the group might reveal the existence of more new species.

This paper aims to unite and discuss all known information on systematics of the New Guinean *Exocelina* provided in our previous studies (Table 1), focusing on the infrageneric structure of the group. Since all species groups were treated in numerous separate publications, we believe that this paper will provide better orientation in this species-rich genus, and easier species identification. Additionally, since the proposed species-group structure is based not only on morphological characters but also supported by molecular analyses, we believe that it is a good tool for understanding New Guinean *Exocelina* phylogeny and character evolution.

## ﻿Materials and methods

Our study is based on published articles on the taxonomy of New Guinean *Exocelina*. In cases where specimen study was necessary, we followed the methods described in detail in our previous articles (Shaverdo et al. 2012, 2014; Shaverdo and Balke 2014).

The results are presented as a species-group table and a key to species groups. The table includes all known species groups of New Guinean *Exocelina* with their numbers of species and subspecies, species-group distribution, and references for each group: species-group diagnoses, keys to species identification and species descriptions. The key provides identification to the species-group level and is meant to be a start point in the determination of New Guinean *Exocelina*. To illustrate the key, figures from our published articles are used, as indicated for each figure in the captions.

## ﻿Results

### ﻿Species-group structure

We recognise 26 species groups of New Guinean *Exocelina*. The groups were proposed based on our study of morphological characters of the species and data from molecular phylogenetic analyses, where the main diagnostic criteria were structure of the genitalia and relative position of the species in the phylogenetic trees.

Most of the species in New Guinea are lotic, that is, associated with running water habitats. All of these species form one monophyletic group and are, thus, endemic to the island. The only exception is the stagnophilous species *E.baliem* Shaverdo, Hendrich & Balke, 2013, which belongs to the *E.ferruginea* group. This group has two other representatives: the Australian *E.ferruginea* (Sharp, 1882) and the New Caledonian *E.inexpectata* Wewalka, Balke & Hendrich, 2010 (Shaverdo et al. 2013).

**Table 1.** Checklist of the species groups of New Guinea *Exocelina*.

N	Species group	Number of spp./subspp.	Species distribution	Reference with species-group diagnosis, key, species descriptions
			IN (Indonesia): Province: Regency	
			PNG (Papua New Guinea): Region: Province	
1	*aipo*	4	**IN**: Papua: Pegunungan Bintang, Yahukimo	Balke (1998); Balke et al. 2007 (as *me*-group); Shaverdo et al. (2017a)
2	*aipomek*	1	**IN**: Papua: Pegunungan Bintang	Balke (1998); Shaverdo et al. (2019)
			**PNG**: Momase: Sandaun	
3	*ascendens*	2	**IN**: Papua: Puncak Jaya, Puncak, Pegunungan Bintang	Balke (1998); Shaverdo et al. (2017b)
4	*bacchusi*	5 / 1	**IN**: Papua: Pegunungan Bintang	Balke (1998); Shaverdo et al. (2019, 2021)
			**PNG**: Highlands: Eastern Highlands, Simbu; Momase: Madang, Morobe; Papua: Central, Gulf	
5	*bagus*	1	**IN**: Papua: Nabire	Balke (1998, 2001); Shaverdo et al. (2017b)
6	*broschii*	5	**PNG**: Highlands: Enga, Eastern and Western Highlands, Hela, Simbu; Momase: Madang, Sandaun; Papua: Gulf	Balke (1998); Shaverdo et al. (2005, 2016c)
7	*casuarina*	24	**IN**: West Papua: Nabire; Papua: Puncak, Jayapura, Pegunungan Bintang	Balke (1998, 1999); Shaverdo et al. (2018)
			**PNG**: Highlands: Eastern, Southern and Western Highlands, Enga, Simbu; Momase: East Sepik, Madang, Morobe, Sandaun	
8	*danae*	15	**IN**: West Papua: Teluk Wondama; Papua: Paniai, Intan Jaya, Puncak Jaya, Puncak, Pegunungan Bintang	Balke (1998); Shaverdo et al. (2016d)
			**PNG**: Highlands: Eastern and Western Highlands, Enga, Simbu; Momase: Madang, Morobe, Sandaun, Papua: Central, Gulf, National Capital District, Oro (Northern), Fly (Western)	
9	*ekari*	62 / 3	**IN**: West Papua: Fak-Fak, Manokwari, Raja Ampat, Sorong, Teluk Wondama; Papua: Jayapura, Mamberamo Raya, Mimika, Nabire, Paniai, Pegunungan Bintang, Sarmi, Yahukimo, Yapen Islands	Balke (1998); Shaverdo and Balke (2019); Shaverdo et al. (2005, 2012, 2014, 2016a, 2020a, b, 2021)
			**PNG**: Highlands: Eastern, Southern and Western Highlands, Enga, Hela, Simbu; Momase: East Sepik, Madang, Morobe, Sandaun; Papua: Gulf, Fly (Western)	
10	*ferruginea* (*E.baliem*)	1	**IN**: Papua: Jayawijaya	Shaverdo et al. (2013)
11	*iratoi*	1	**IN**: Papua: Puncak	Shaverdo et al. (2017b)
12	*jaseminae*	4	**PNG**: Highlands: Eastern Highlands; Momase: Morobe; Papua: Central	Balke (1998); Shaverdo et al. (2019)
13	*koroba*	1	**PNG**: Highlands: Hela	Shaverdo et al. (2019)
14	*larsoni*	3	**PNG**: Highlands: Eastern Highlands, Simbu; Momase: Madang; Papua: Central, National Capital	Balke (1998); Shaverdo et al. (2019)
15	*likui*	1	**IN**: Papua: Puncak Jaya	Shaverdo et al. (2017b)
16	*mekilensis*	1	**PNG**: Momase: Sandaun	Shaverdo et al. (2019)
17	*monae*	1	**PNG**: Momase: Morobe	Balke (1998)
18	*morobensis*	1	**PNG**: Momase: Morobe	Shaverdo et al. (2019)
19	*okbapensis*	4 / 1	**IN**: Papua: Jayawijaya, Pegunungan Bintang, Yahukimo	Shaverdo et al. (2017a)
			**PNG**: Momase: Sandaun	
20	*pui*	1	**IN**: Papua: Puncak	Shaverdo et al. (2017b)
21	*ransikiensis*	1	**IN**: West Papua: Manokwari; Papua: Nabire	Shaverdo et al. (2016b, 2017b)
22	*skalei*	2	**IN**: West Papua: Kaimana; Papua: Mimika	Shaverdo et al. (2020b)
23	*takime*	2	**IN**: Papua: Pegunungan Bintang	Balke (1998); Shaverdo et al. (2019)
			**PNG**: Momase: Sandaun	
24	*ullrichi*	3	**PNG**: Highlands: Eastern Highlands; Momase: Morobe	Balke (1998); Shaverdo and Balke (2014)
25	*warasera*	4	**PNG**: Highlands: Eastern Highlands, Simbu; Momase: Morobe; Papua: Central	Shaverdo et al. (2019)
26	*wigodukensis*	2	**IN**: Papua: Puncak Jaya	Shaverdo et al. (2017b)

### ﻿Key to New Guinean species groups of *Exocelina*

The key is proposed for identification of the species groups and species in the case of monotypic groups. The keys to species of individual groups can be found in the publications listed in Table 1.

The key is mostly based on male characters, but organised in a way to get one as far as possible with female identification. In many cases, females cannot be assigned to species due to the similarity of their external and internal structures (for female genitalia see figs 17a, b in Shaverdo et al. (2005) and fig. 7 in Shaverdo et al. (2013)). Some species are rather similar in external morphology and, therefore, in most cases, the male genitalia need to be studied for reliable species identification. However, for some groups, identification of the females is possible to the species group and even to species. The important point here is not to separate females from males from the same locality. Their identification should follow identification of the males of all species from the chosen locality. If co-occurring species are not numerous (2–4 species), successful identifications of females are highly possible.

**Table:** 

1	Elytron covered with short longitudinal strioles (Fig. 1)	***ferruginea* group (*E.baliem*)**
–	Elytron without strioles	**2**
2	Pronotum with lateral bead, rarely narrow but distinct	**3**
–	Pronotum without lateral bead, sometimes (especially in females) with bead traces or even narrow bead, in this case several specimens of population should be checked	**22**
3	Male protarsomere 5 strongly modified: concave ventrally, sometimes with anteroproximal setae enlarged. Male protarsomere 4 with anterolateral hook-like seta small, not developed (Fig. 2). Male antennomeres modified	***aipo* group**
–	Male protarsomere 5 not modified. Male protarsomere 4 with anterolateral hook-like seta small to large (Fig. 3). Male antennomeres modified or not	**4**
4	Median lobe of aedeagus with discontinuous outline in ventral and often in lateral views (Fig. 4)	***ekari* group (in part)**
–	Median lobe of aedeagus with continuous or slightly discontinuous apically outline in ventral view	**5**
5	Paramere with most of setae very short, inconspicuous, some distal setae stronger. Median lobe without setae, with continuous or slightly discontinuous apically outline in ventral view (Fig. 5A, B)	***skalei* group**
–	Paramere with strong and long distal setae, rarely with all setae very short, inconspicuous. Median lobe with or without setae, with continuous outline	**6**
6	Median lobe with fork-like apex of ventral sclerite (Fig. 6A, B)	***broschii* group**
–	Median lobe with apex of ventral sclerite more or less deeply separated in two (rarely three) lobes (Fig. 6C, D)	**7**
7	Male antennomere 2 distinctly larger than other antennomeres (Fig. 7)	**8**
–	Male antennomeres simple or differently modified	**9**
8	Paramere with very short, inconspicuous setae. Median lobe with minuscule tip of apex curved upwards in lateral view (Fig. 8A)	***ullrichi* group**
–	Paramere with long, distinct setae. Median lobe with broadly pointed apex in lateral view (Fig. 8B)	***danae* group (*miriae* subgroup)**
9	Median lobe in ventral view with distinctly concave apex forming two apical lobes	**10**
–	Median lobe in ventral view pointed, truncate, or rounded, without two apical lobes	**11**
10	Median lobe long and slender, with fine apical setae; its apical lobes narrow and concave in lateral view (Fig. 9A)	***monae* group**
–	Median lobe shorter and more robust, without setae; its apical lobes broader, usually rounded in lateral view (Fig. 9B)	***jaseminae* group**
11	Median lobe very broad, robust, almost parallel-sided, with weak median constriction in ventral view; lateral sides strongly thickened; apexes of ventral sclerites very unequal: right one much longer than left one (Fig. 10)	***larsoni* group**
–	Median lobe slender and of different shape; lateral sides not or only slightly thickened; apexes of ventral sclerites equal or slightly unequal in length	**12**
12	Median lobe with setae	**13**
–	Median lobe without setae	**14**
13	Beetle larger, TL–H 5.3–5.8 mm	***ascendens* group (in part: *E.ascendens*)**
–	Beetle smaller, TL–H 3.4–4.75 mm	***danae* group (in part)**
14	Paramere with distinct dorsal notch and subdistal part well developed (Fig. 11A)	**15**
–	Paramere without dorsal notch, slightly concave, subdistal part not evidently separated (Fig. 11B)	**18**
15	Subdistal part of paramere large, long, with numerous strong setae	**16**
–	Subdistal part of paramere small, with a tuft of setae	**17**
16	Pronotum with distinct lateral bead. Median lobe longer and slender; lateral sides not thickened; in ventral view, narrow, slightly tapering to narrowly rounded apex; in lateral view, apex thin and elongate (Fig. 12A)	***aipomek* group (*E.aipomek*)**
–	Pronotum with narrow lateral bead. Median lobe shorter and more robust, lateral sides slightly thickened; in ventral view, broadened medially or subdistally, apex broadly pointed or slightly concave; in lateral view, apex thicker, not elongate (Fig. 12B)	***takime* group**
17	Median lobe robust, apex with strong, short prolongation, curved downwards in lateral view (Fig. 13A). Subdistal part of paramere larger	***koroba* group (*E.koroba*)**
–	Median lobe slender, evenly curved, apex without apical prolongation, very slightly curved downwards in lateral view (Fig. 13B). Subdistal part of paramere smaller	***okbapensis* group**
18	Paramere with dorsal setae divided into distinct, evidently stronger subdistal setae and inconspicuous proximal ones due to much weaker median setation (Fig. 14A)	**19**
–	Paramere with dorsal setae uniform, inconspicuous or distinct, or with proximal setae distinct and long, sometimes stronger than subdistal (Fig. 14B)	**21**
19	Median lobe almost parallel-sided, often narrowed distally before or to apex or broadened subdistally; its apex usually with thickened sides, slightly or distinctly enlarged (“swollen”, often in shape of a baby pacifier), rounded, truncate, or slightly concave in ventral view (Fig. 15)	***casuarina* group (in part)**
–	Median lobe different. Apex without such modifications	**20**
20	Median lobe thinner in apical half; in ventral view, evenly attenuated to pointed apex and, in lateral view, evenly broad, with rounded apex; its lateral margins slightly thickened (Fig. 16A)	***morobensis* group (*E.morobensis*)**
–	Median lobe more robust; evenly attenuated to bluntly pointed apex in ventral and lateral views; lateral margins not thickened, right one can be slightly concave distally in lateral view (Fig. 16B)	***warasera* group**
21	Median lobe in lateral view slender, almost straight, only apex distinctly curved downwards; in ventral view, with apex very broadly rounded (Fig. 16C). Setae of paramere very fine, inconspicuous. Beetle dorsally matt, with distinct to strong punctation and microreticulation	***ransikiensis* group (*E.ransikiensis*)**
–	Median lobe in lateral view broader, more strongly curved, more or less evenly attenuated to thinner apex; in ventral view, apex bluntly pointed (Fig. 16D). Setae of paramere more distinct. Dorsal surface sculpture different, usually very fine	***bacchus* group**
22	Median lobe with discontinuous outline in ventral and often in lateral views (Fig. 4)	***ekari* group (in part)**
–	Median lobe with continuous outline	**23**
23	Male antennomeres extremely modified: antennomeres 4–6 excessively large, 3 and 7 strongly enlarged (Fig. 17)	***bagus* group (*E.bagus*)**
–	Male antennomeres simple or slightly enlarged	**24**
24	Apex of median lobe with two lateral and one dorsal prolongations (Fig. 18)	***iratoi* group (*E.iratoi*)**
–	Apex of median lobe without such modifications	**25**
25	Paramere with numerous small spines, no long setae. Apex of median lobe thick, short and slightly curved downwards, its minuscule tip curved upwards in lateral view (Fig. 19)	***mekilensis* group (*E.mekilensis*)**
–	Paramere with long setae. Apex of median lobe pointed or rounded, without such modifications	**26**
26	Median lobe with distinct subapical setae	**27**
–	Median lobe without setae, in some species with minuscule spines	**28**
27	Beetle larger, TL–H > 4.5 mm. Apex of median lobe pointed in lateral view and rounded in ventral view (Fig. 20A)	***ascendens* group (in part: *E.tomhansi*)**
–	Beetle smaller, TL–H < 3.6 mm. Apex of median lobe roundly truncate in lateral view and concave in ventral view (Fig. 20B)	***pui* group (*E.pui*)**
28	Apex of median lobe with thickened sides, often distinctly enlarged (“swollen”), in lateral and ventral views often of shape of a baby pacifier, rounded, truncate, or slightly concave in ventral view (Fig. 20C, D)	***casuarina* group (in part)**
–	Apex of median lobe of different shape, relatively thin, elongate in lateral view and broadly truncate in ventral view	**29**
29	Beetle larger, TL–H 3.7–4.35 mm. Male antennomeres enlarged. Median lobe longer. Paramere with numerous small and few large proximal setae; large setae with basal prolongations (Fig. 21A)	***wigodukensis* group**
–	Beetle smaller, TL–H 3.2–3.6 mm. Male antennomeres simple. Median lobe shorter. Paramere only with small proximal setae (Fig. 21B)	***likui* group (*E.likui*)**

**Figures 1–3.**
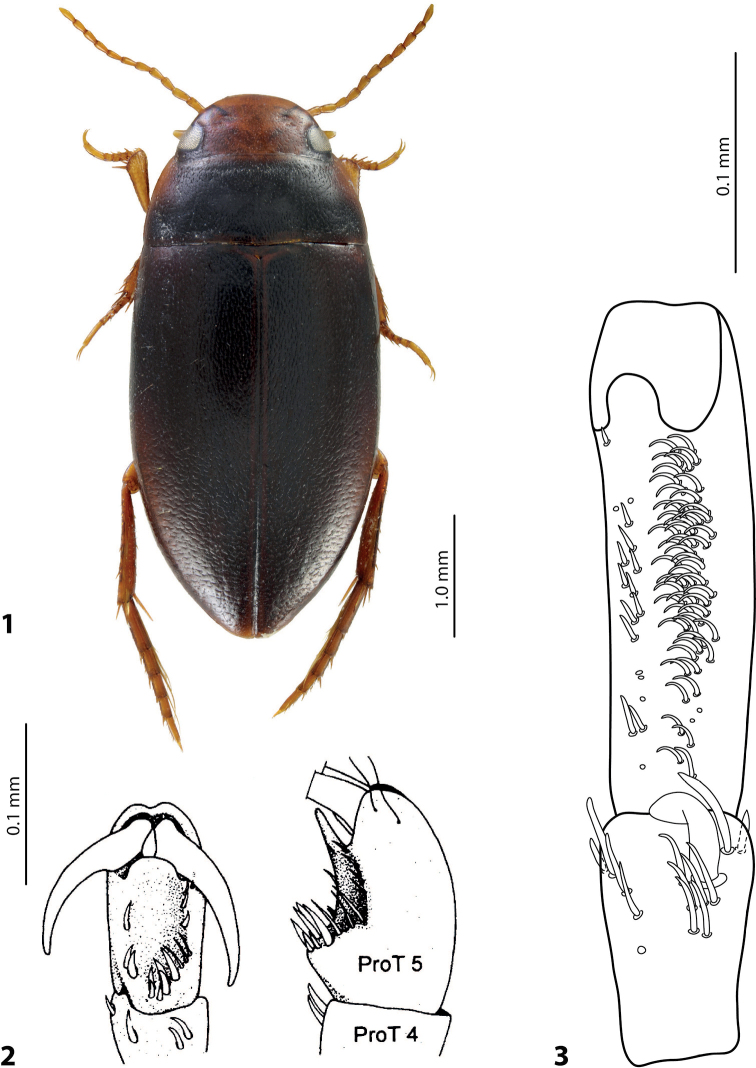
**1** Habitus of *Exocelinabaliem* Shaverdo, Hendrich & Balke, 2013, female (Shaverdo et al. 2013: 86, fig. 1) **2** Structure of male protarsomeres 4 and 5 of *E.aipo* (Balke, 1998) in ventral and lateral views (Balke 1998: 319, fig. 25) **3** Male protarsomeres 4 and 5 of *E.mimika* Shaverdo & Balke, 2020 in ventrolateral view (Shaverdo et al. 2020b: 140, fig. 9B).

**Figures 4, 5.**
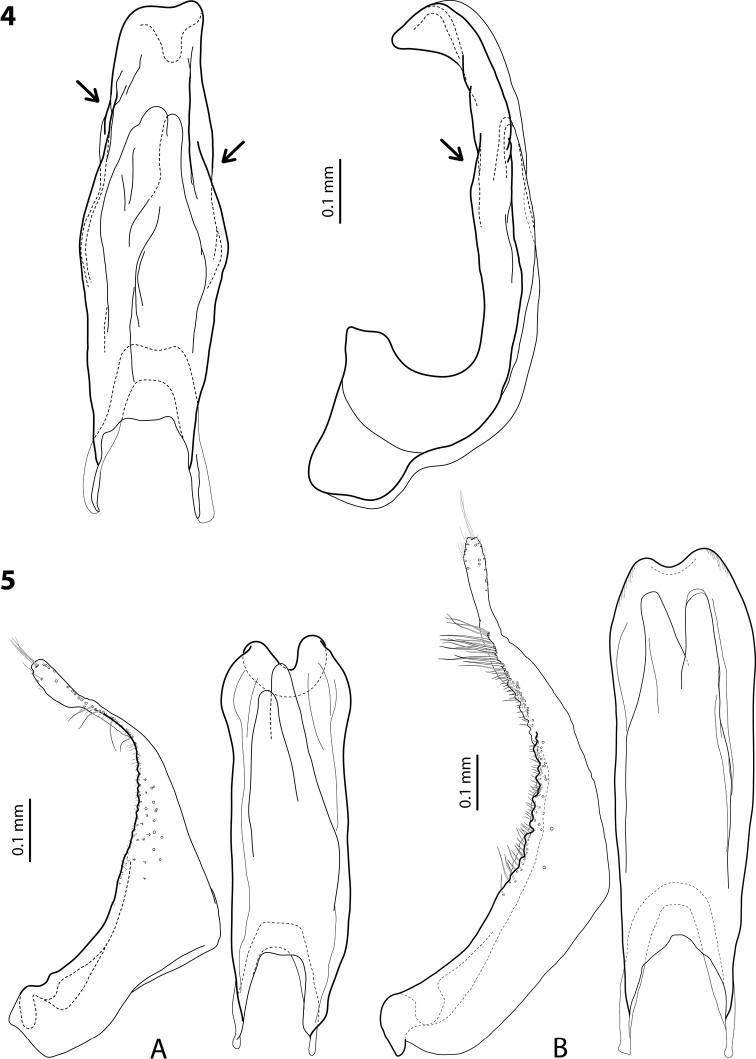
**4** Discontinuous outlines (see arrows) of median lobe of aedeagus of *Exocelinaoceai* Shaverdo, Hendrich & Balke, 2012 in ventral and lateral views (Shaverdo et al. 2012: 46, fig. 1) **5** Paramere and median lobe in ventral view of **A***E.skalei* Shaverdo & Balke, 2014 (Shaverdo et al. 2014: 51, fig. 1C, D) **B***E.mimika* Shaverdo & Balke, 2020 (Shaverdo et al. 2020b: 140, fig. 9C, A).

**Figure 6.**
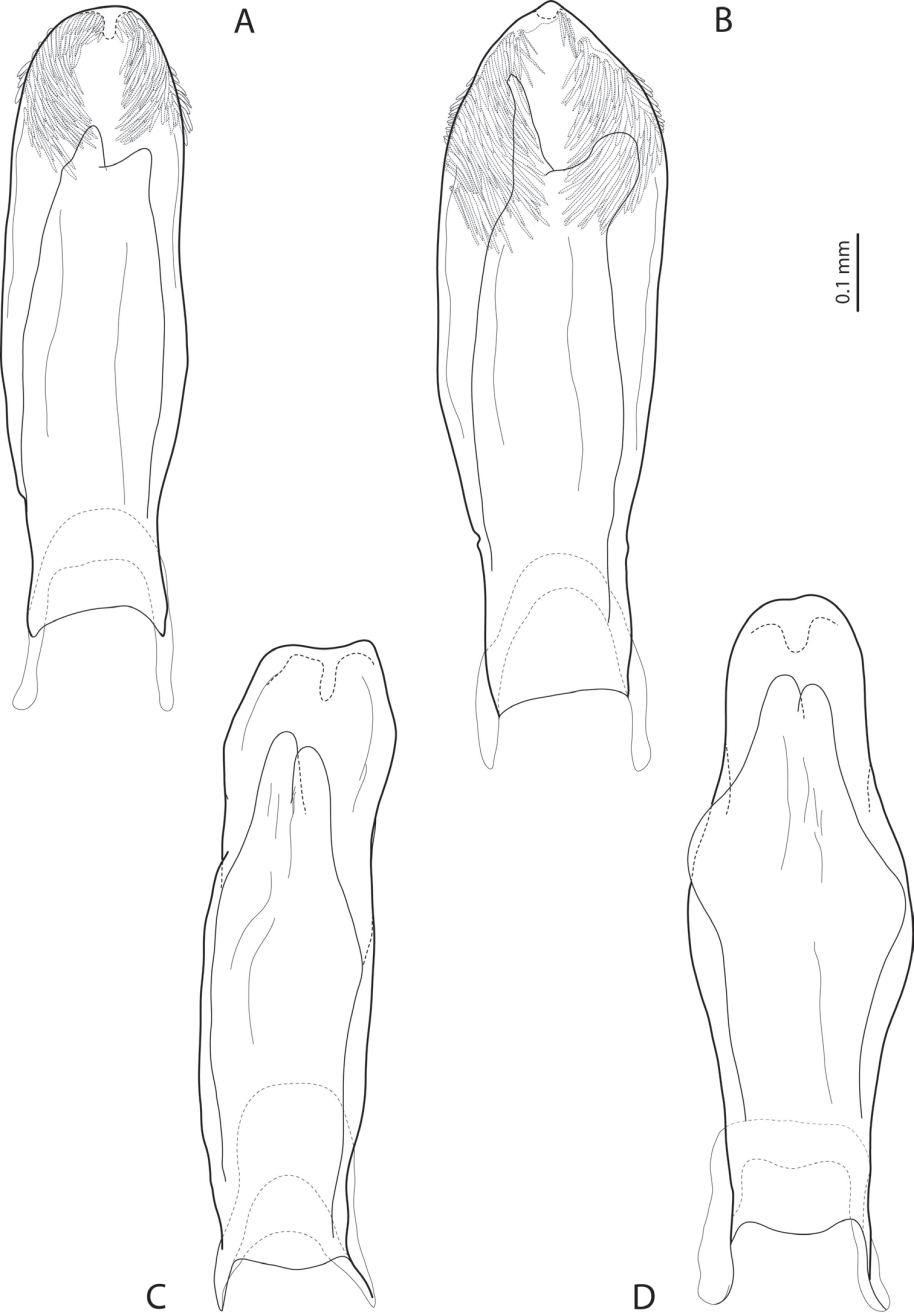
Median lobe in ventral view of **A***Exocelinabroschii* (Balke, 1998) (Shaverdo et al. 2016c: 134, fig. 8B) **B***E.mondmillensis* Shaverdo, Sagata & Balke, 2016 (Shaverdo et al. 2016c: 139, fig. 11B) **C***E.gorokaensis* Shaverdo & Balke, 2014 (Shaverdo et al. 2014: 63, fig. 14C) **D***E.ksionseki* Shaverdo & Balke, 2014 (Shaverdo et al. 2014: 67, fig. 18C).

**Figures 7, 8.**
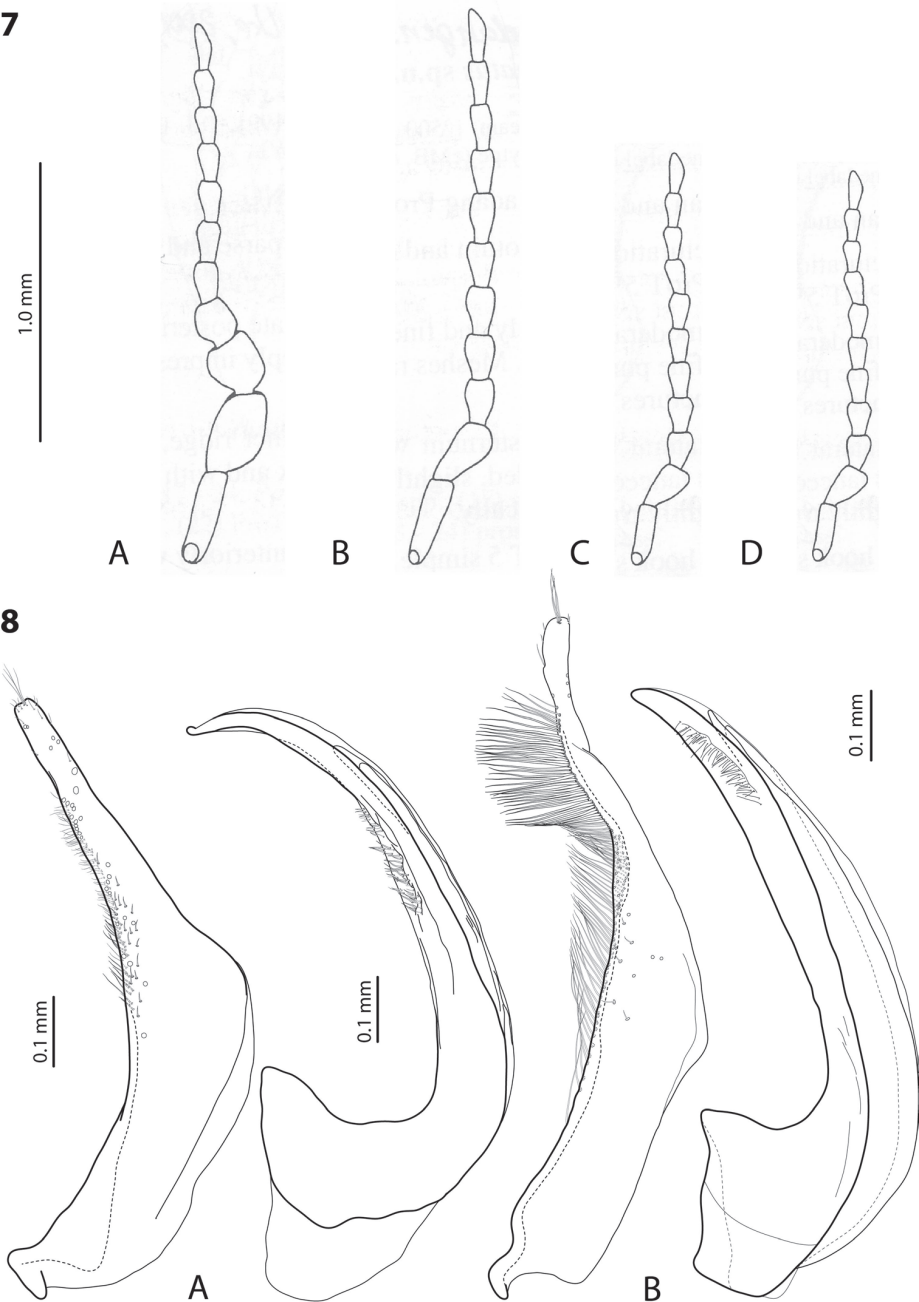
**7** Male antennae of **A***Exocelinakainantuensis* (Balke, 2001) **B***E.ullrichi* (Balke, 1998) **C***E.miriae* (Balke, 1998) **D***E.rufa* (Balke, 1998) (Balke 1998: 315, figs 12–15) **8** Paramere and median lobe in lateral view of **A***E.kinibeli* Shaverdo & Balke, 2014 (Shaverdo and Balke 2014: 35, fig. 3; p. 36, fig. 4) **B***E.miriae* (Shaverdo et al. 2016d: 81, fig. 2C, B).

**Figures 9–11.**
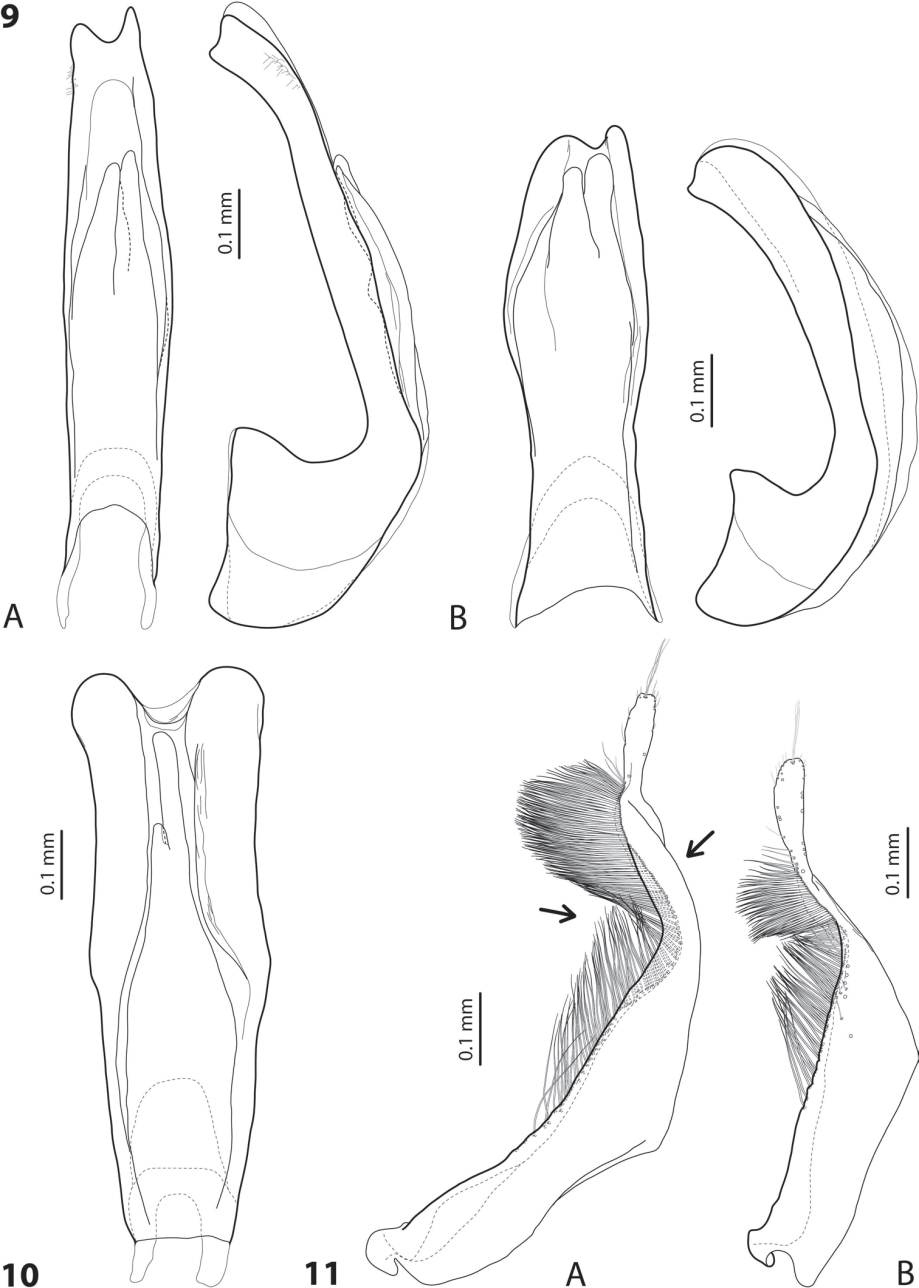
**9** Median lobe in ventral and lateral views of **A***Exocelinamonae* (Balke, 1998) **B***E.jaseminae* (Balke, 1998) (Shaverdo et al. 2019: 114, fig. 31A, B) **10** Median lobe in ventral view of *E.larsoni* (Balke, 1998) (Shaverdo et al. 2019: 126, fig. 40B) **11** Paramere of **A***E.aipomek* (Balke, 1998) (Shaverdo et al. 2019: 79, fig. 5C) **B***E.casuarina* (Balke, 1998) (Shaverdo et al. 2018: 54, fig. 26C).

**Figure 12.**
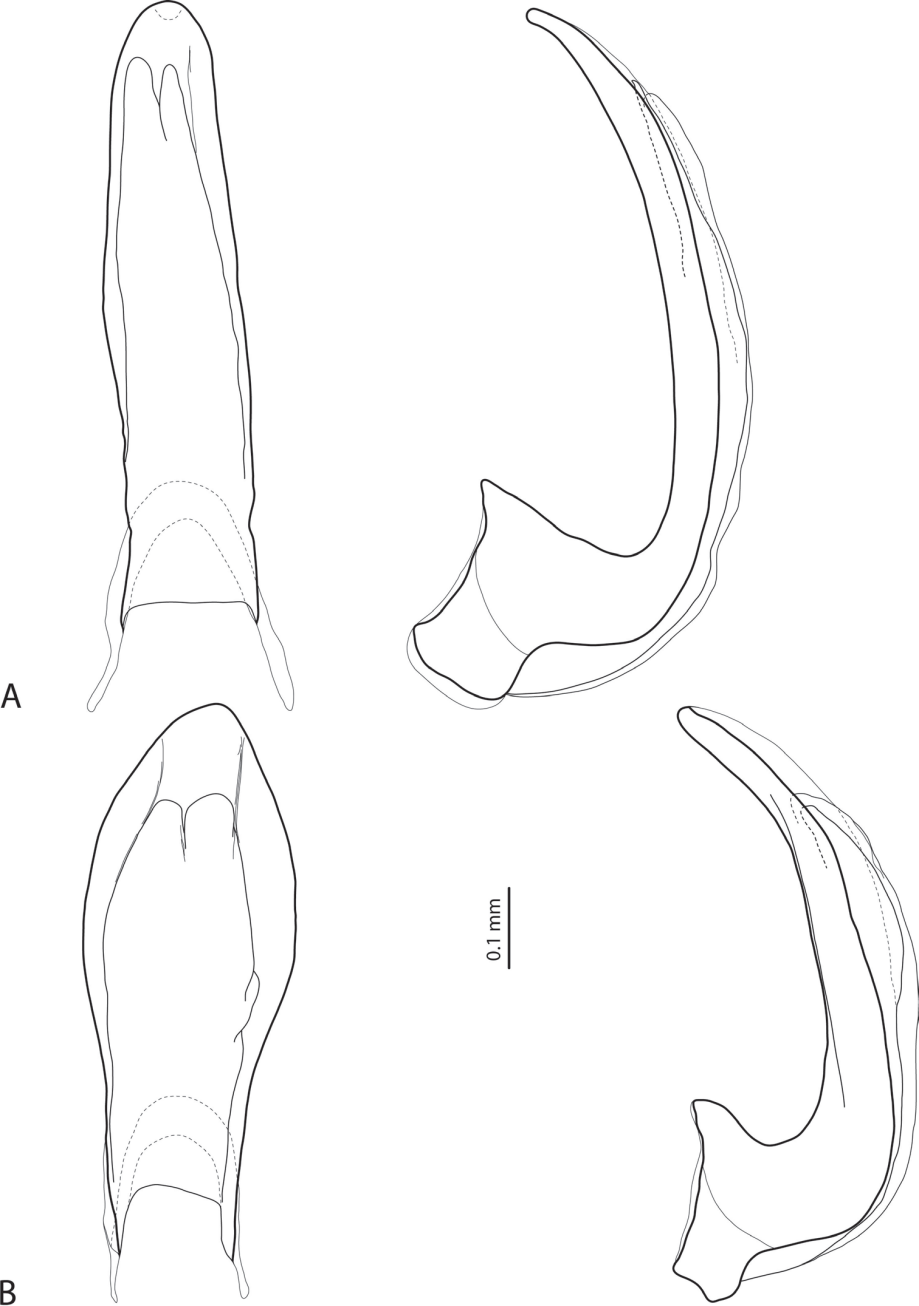
Median lobe in ventral and lateral views of **A***Exocelinaaipomek* (Balke, 1998) (Shaverdo et al. 2019: 79, fig. 5A, B) **B***E.takime* (Balke, 1998) (Shaverdo et al. 2019: 131, fig. 44A, B).

**Figure 13.**
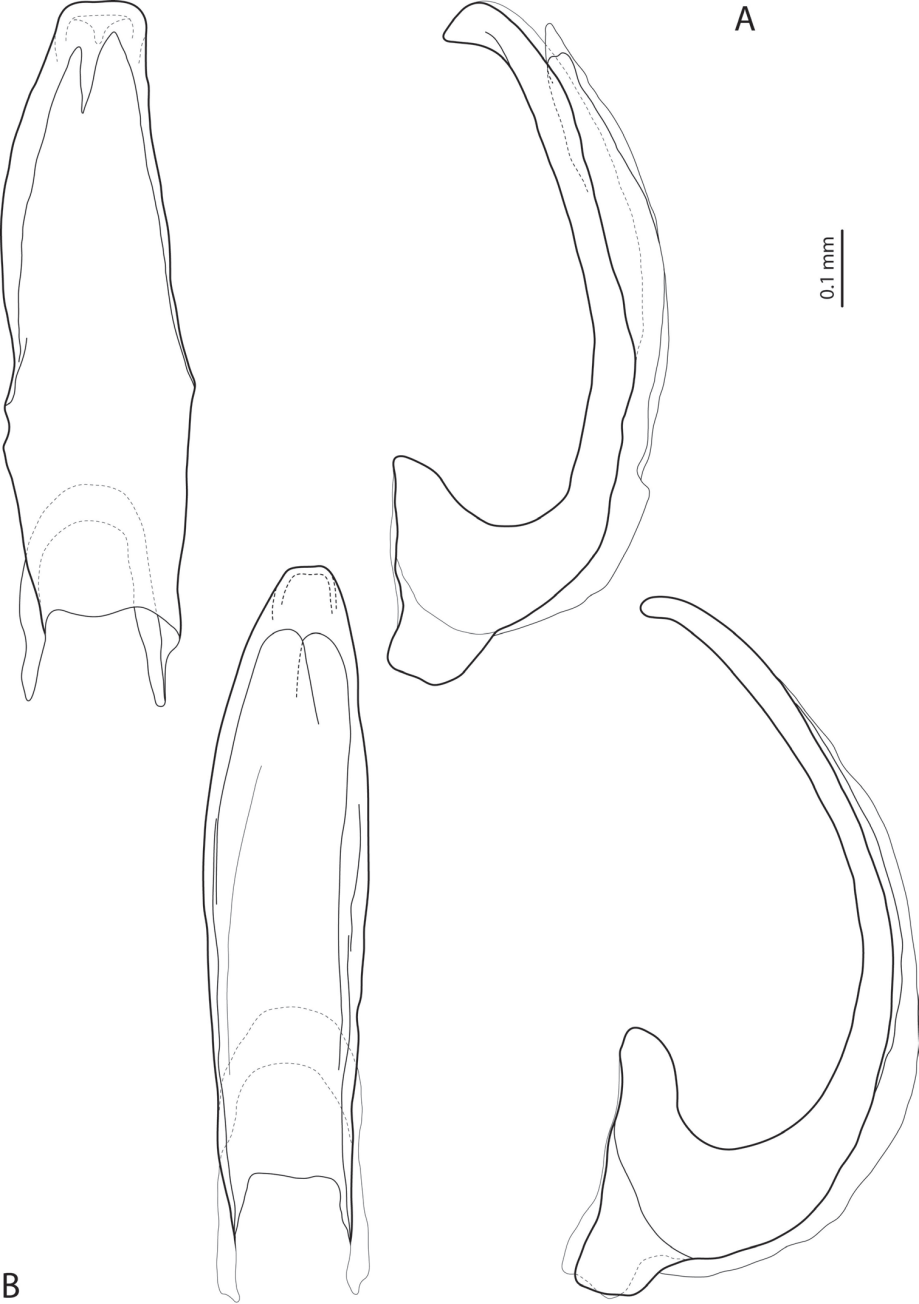
Median lobe in ventral and lateral views of **A***Exocelinakoroba* Shaverdo & Balke, 2019 (Shaverdo et al. 2019: 82, fig. 8B, C) **B***E.okbapensis* Shaverdo & Balke, 2017 (Shaverdo et al. 2017a: 23, fig. 5B, C).

**Figures 14–15.**
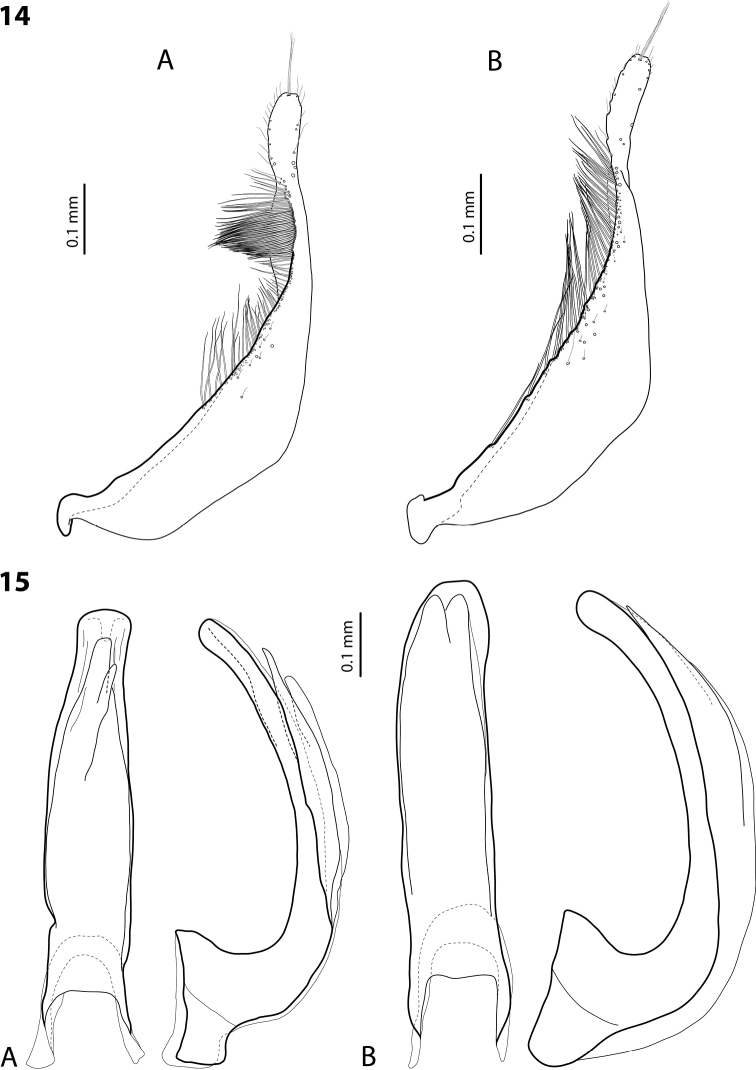
**14** Paramere of **A***Exocelinapseudopusilla* Shaverdo & Balke, 2018 (Shaverdo et al. 2018: 63, fig. 42C) **B***E.bacchusi* (Balke, 1998) (Shaverdo et al. 2019: 104, fig. 22C) **15** Median lobe in ventral and lateral views of **A***E.sumokedi* Shaverdo & Balke, 2018 (Shaverdo et al. 2018: 58, fig. 34A, B) **B***E.desii* (Balke, 1998) (Shaverdo et al. 2018: 61, fig. 39A, B).

**Figure 16.**
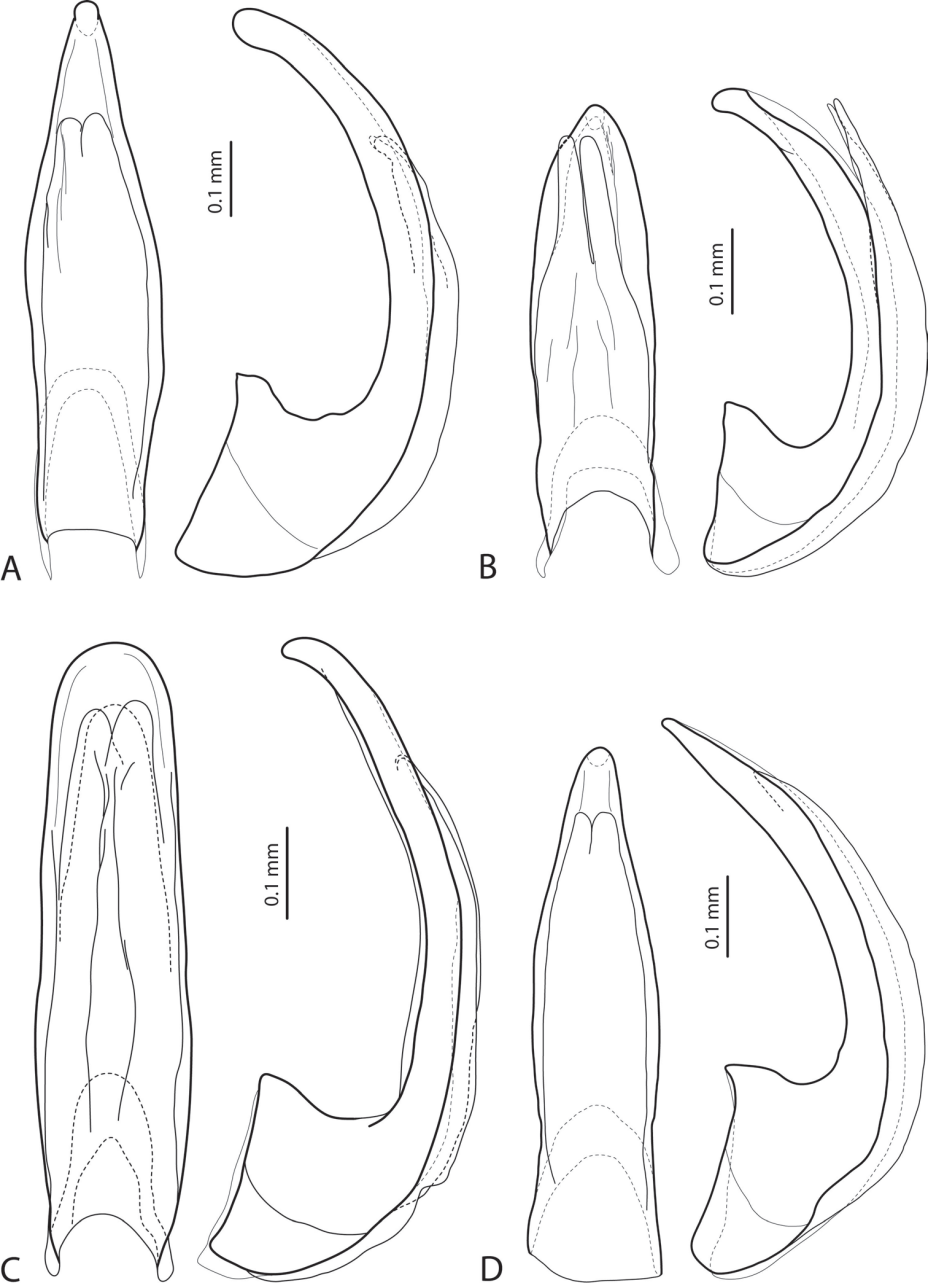
Median lobe in ventral and lateral views of **A***Exocelinamorobensis* Shaverdo & Balke, 2019 (Shaverdo et al. 2019: 88, fig. 10A, B) **B***E.warasera* Shaverdo & Balke, 2019 (Shaverdo et al. 2019: 140 fig. 52A, B) **C***E.ransikiensis* Shaverdo, Panjaitan & Balke, 2016 (Shaverdo et al. 2016b: 106, figs 4, 5) **D***E.bacchusi* (Balke, 1998) (Shaverdo et al. 2019: 104, fig. 22A, B).

**Figures 17–19.**
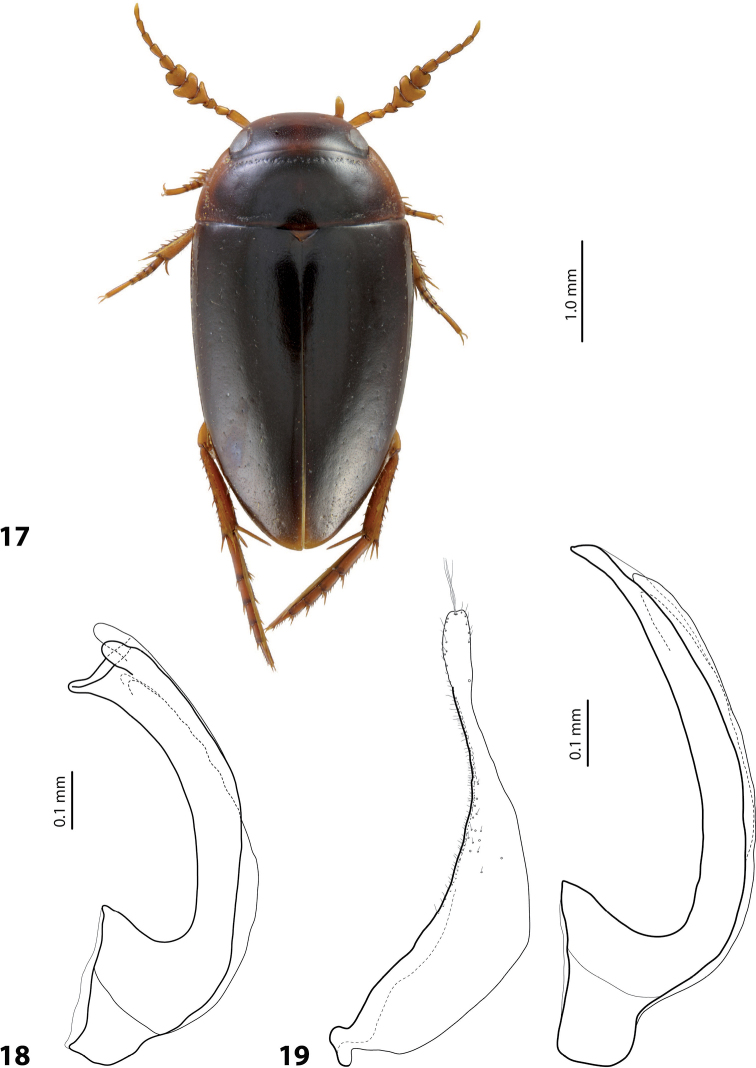
**17** Habitus of *Exocelinabagus* (Balke & Hendrich, 2001) (Shaverdo et al. 2017b: 111, fig. 6) **18** Median lobe in lateral view of *E.iratoi* Shaverdo & Balke, 2017 (Shaverdo et al. 2017b: 115, fig. 13B) **19** Paramere and median lobe in lateral view of *E.mekilensis* Shaverdo & Balke, 2019 (Shaverdo et al. 2019: 85, fig. 9C, B).

**Figure 20.**
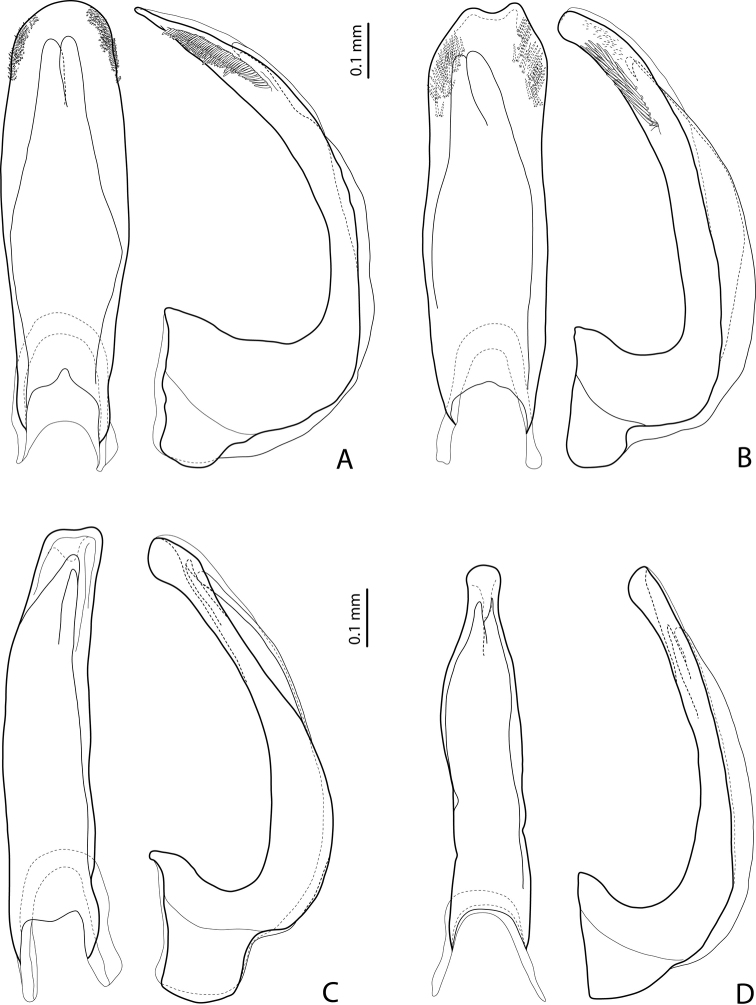
Median lobe in ventral and lateral views of **A***Exocelinatomhansi* Shaverdo & Balke, 2017 (Shaverdo et al. 2017b: 114, fig. 12A, B) **B***E.pui* Shaverdo & Balke, 2017 (Shaverdo et al. 2017b: 116, fig. 16A, B) **C***E.casuarina* (Balke, 1998) (Shaverdo et al. 2018: 54, fig. 26A, B) **D***E.keki* Shaverdo & Balke, 2018 (Shaverdo et al. 2018: 56, fig. 30A, B).

**Figure 21.**
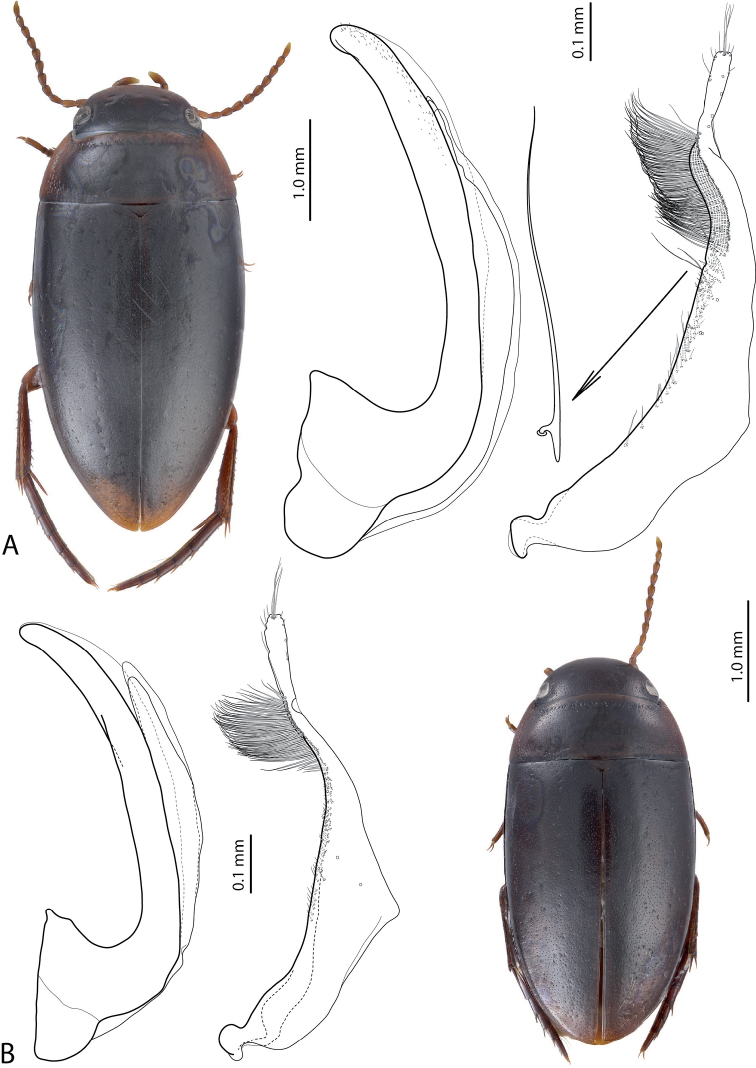
Habitus, median lobe in lateral view and paramere of **A***Exocelinapulukensis* Shaverdo & Balke, 2017 (Shaverdo et al. 2017b: 112, fig. 10; 117, fig. 18B, C) **B***E.likui* Shaverdo & Balke, 2017 (Shaverdo et al. 2017b: 112, fig. 7; 116, fig. 15A, B).

### ﻿Phylogeny and infrageneric structure

**Figure 22.**
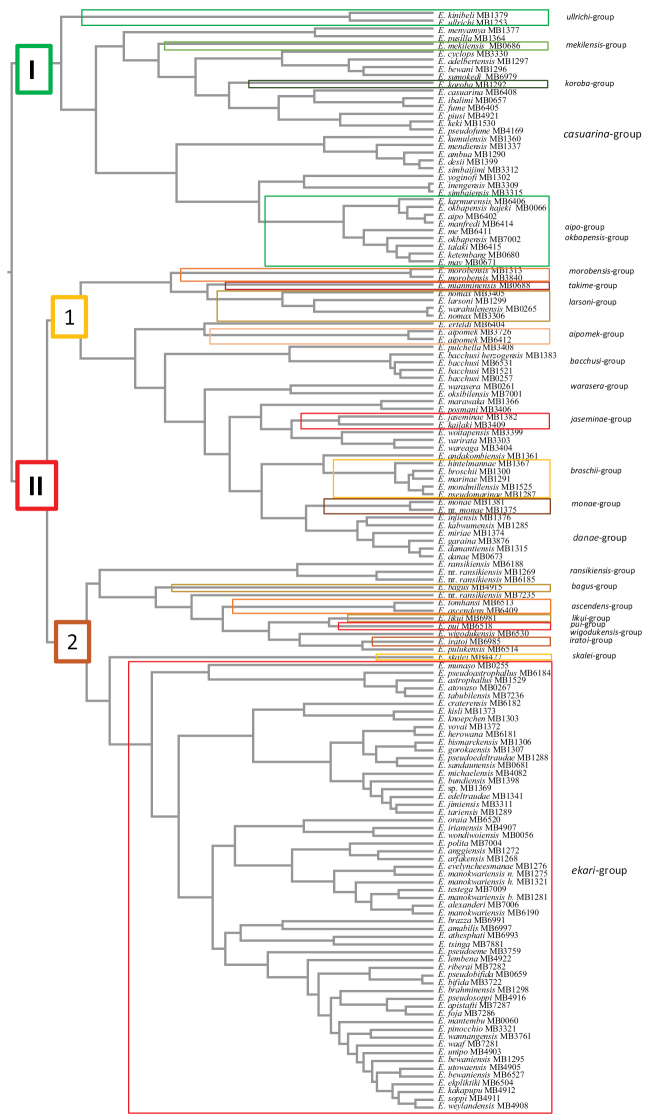
Phylogenetic relationships and species-group structure of *Exocelina* species of New Guinea. Monophyletic groups are highlighted. *Exocelinabaliem* Shaverdo, Hendrich & Balke, 2013 is excluded.

The infrageneric structure of New Guinean *Exocelina* is largely based on the molecular phylogeny of the group, most of the species groups being represented as monophyletic clades on the phylogenetic tree (Fig. 22). We consider this approach to be very useful for understanding the taxonomy and evolution of such a species-rich group.

Earlier phylogenetic analyses based on molecular data substantiated the lotic New Guinean *Exocelina* as a monophyletic group, which emerged from a single colonization event by an Australian lineage and led to a rich species radiation on the island (Balke et al. 2004, 2007). More recent investigations suggested an origin of New Guinean *Exocelina* during the late Miocene, ca 5 or 9 million years ago (Toussaint et al. 2014, 2015), or even in the mid-Miocene, ca 17 Ma, when the New Guinean orogeny was at an early stage (Toussaint et al. 2021) and inferred a constant process of lineage diversification with a continuous slowdown in speciation.

**Figure 23.**
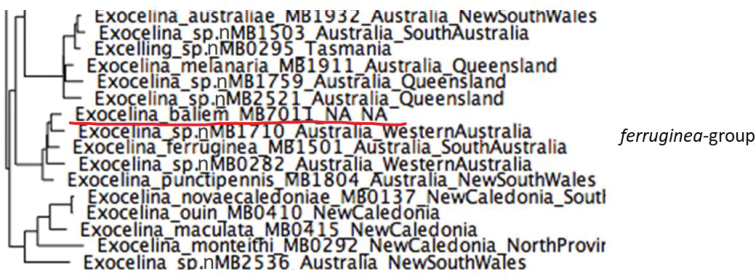
Phylogenetic position of *Exocelinabaliem* Shaverdo, Hendrich & Balke, 2013 amongst Australian species in the *E.ferruginea* group.

A second colonization event was by a lentic species, evident from the presence of only one extant species, i.e., *E.baliem* from wetlands in the Baliem Valley of Papua Province (Shaverdo et al. 2013). According to an unpublished molecular phylogenetic analysis, this species forms a clade with the Australian *E.ferruginea* and the New Caledonian *E.inexpectata* and is placed together with them in the *E.ferruginea* group (Fig. 23).

The 151 lotic New Guinean *Exocelina* species form a monophyletic group, which contains two clades: the smaller clade I with only six species groups and the distinctly larger clade II with 19 species groups (Fig. 22). In clade I, only the monophyly of the *E.ullrichi* group and two monotypic groups (*E.mekilensis* group and *E.koroba* group) is well resolved. The majority of the remaining species are placed in the *E.casuarina* group, whose phylogeny is discussed in details in Shaverdo et al. (2018). With 24 species, this group is the second largest species group of New Guinean *Exocelina*. Interestedly, the *E.aipo* and *E.okbapensis* groups together form a monophyletic clade despite having rather distinct morphologies.

Clade II itself also consists of two large subclades (1 and 2 in Fig. 22). Subclade 1 is very heterogeneous and includes 10 species groups (all species-poor); seven represented as monophyletic clades. The *E.danae* group, the most speciose group of the clade, is inferred as polyphyletic, and the *E.bacchus* and *E.warasera* groups are both paraphyletic. Subclade 2 is the most species-rich clade since it contains the largest species group of New Guinean *Exocelina*, the *E.ekari* group. This group includes 62 species and is monophyletic, forming a monophyletic clade with the *E.skalei* group. The remaining seven groups also form a monophyletic clade (the *E.ascendens* complex) and represent rather different morphological lineages. Whilst species placement into species groups using morphology worked well for the other groups and was later confirmed by phylogenetic analysis, species of the *E.ascendens* complex can mainly be placed using molecular data. Without these data, the groups would probably never be organised in a way that reflects their evolutionary history (see below).

### ﻿Notes on character evolution

More than 20 different morphological characters were used to describe species and organise species into a species-group structure. Some characters are very diverse and have more than 10 different states, e.g., antennal shape (9 states), shape of the median lobe (14 states), setation of the dorsal side of the parameres (12 states). Here, we briefly discuss characters, which we think are the most taxonomically and phylogenetically important and worthy of further study, not only as separate characters but also in combination.

#### Structure of the male genitalia

The shape of the median lobe and paramere and their setation are very diverse and serve as the basic characters for species-group structure in New Guinean *Exocelina*. These characters were primarily used to group the species. The most divergent on these characters is the *E.ekari* group with a discontinuous outline of the median lobe, which could be considered an autoapomorphic character; only *E.skalei* with its slight apical discontinuity of the median lobe belongs to the *E.skalei* group. Together with the recently described *E.mimika* Shaverdo & Balke, 2020, this former member of the *E.ekari* group, was placed into the *E.skalei* group based on the reduced setation of its paramere. Representatives of *E.ekari* group have the most complicated and diverse shape and setation of the median lobe and paramere of all New Guinean *Exocelina*. Most likely, this results from strong sexual selection, or adaptive evolution for sexual isolation, since many species of the group co-occur (up to six species) which is not the case in other species groups.

Almost every species group has its own characteristic shape of the median lobe and paramere and their setation or combination of these characters. As already mentioned above, the most problematic was the placement of species of the *E.ascendens* complex that have male genitalia similar to some species of the *E.casuarina*-, *E.aipo*- and *E.okbapensis* groups.

#### Lateral bead of the pronotum

**Figure 24.**
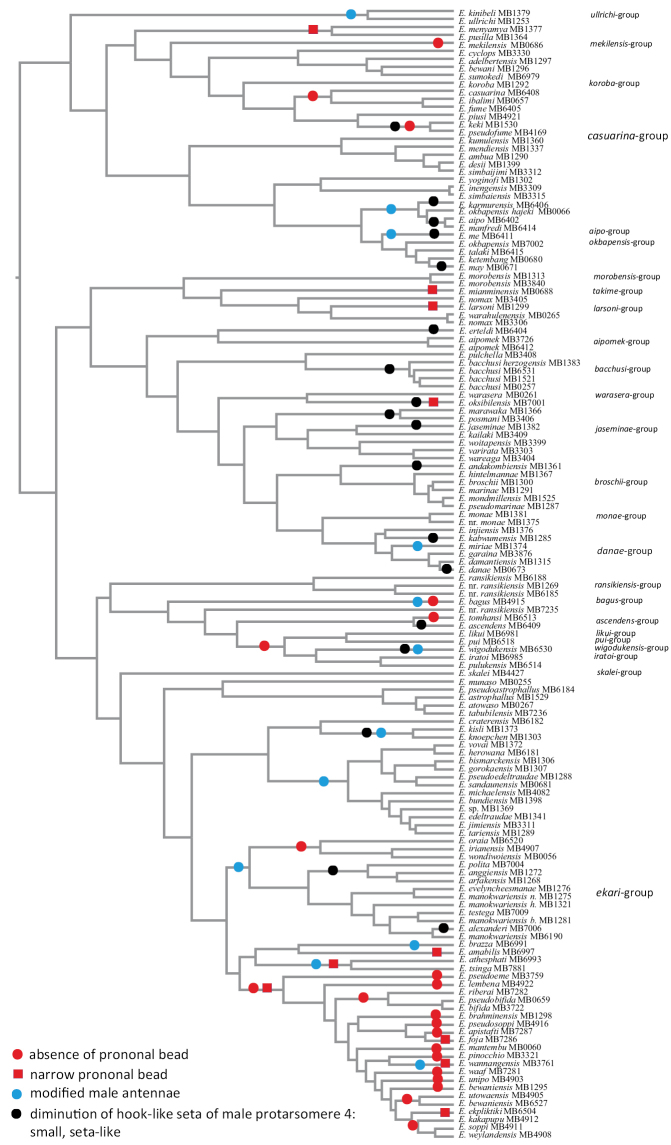
Allocation of four more significant morphological characters amongst *Exocelina* species of New Guinea.

Presence and part or complete reduction of the lateral pronotal bead are states of this actively used in the key character. It is helpful for species identification, but could not be used reliably for phylogenetic purposes. Absence of the lateral pronotal bead is obviously homoplastic. It has developed independently probably up to eight times within New Guinean *Exocelina* (Fig. 24). Interestingly, absence of the lateral pronotal bead is characteristic for some representatives of the largest species groups: *E.ekari* group and *E.casuarina* group. A few species demonstrate a very narrow pronotal lateral bead or presence of its traces.

#### Modification of the male antennae

New Guinean *Exocelina* includes more species with modified antennae than any other genus of Dytiscidae; 45 species have them, mainly in males. The degree of modification and number of antennomeres involved are specific for certain species and/or species groups and strongly vary (up to nine different character states) from almost all antennomeres slightly stout to some of them extravagantly enlarged or extremely reduced (Fig. 17). Half of the species (31 spp.) of the *E.ekari* group have modified antennae, whilst this character is absent in the second largest group, the *E.casuarina* group. For the *E.ullrichi* group, it is a group-diagnostic character, as well as for the *E.miriae* subgroup of the *E.danae* group (Fig. 7). Modified male antennae evolved independently up to 10 times in different groups, including five different lineages within the *E.ekari* group (Fig. 24) and could be used for delimitation of the subgroups within it. It is worth noting that, in some species, modification of the antennae is correlated with stronger dorsal surface structure (especially in females) or/and sometimes with diminution of the hook-like setae of the male protarsomere 4. This may indicate association with sexual processes.

#### Anterolateral seta of the male protarsomere 4

Hook-like anterolateral seta of male protarsomere 4 is the main diagnostic character of the genus *Exocelina* and its unique morphological autoapomorphy. However, secondary diminution or differences in shape are observed in many New Guinean species of the different species groups and have obviously independently involved (Fig. 24). It is currently impossible to postulate why certain species show such characters, although as with other features, sexual selection is likely involved. In the *E.ekari* group, diminution of the seta often occurs in species with enlarged male antennomeres and sometimes also with stronger dorsal surface structure, e.g., species close to *E.polita* (Sharp, 1882). In the *E.casuarina*-, *E.danae*-, *E.jaseminae*-, *E.warasera*-, or *E.bacchusi* groups, representatives of which have simple antennae, reduction of the hook-like seta does not correlate with this character, however, and can be found in species with shiny or matt dorsal surfaces. Although all representatives of the *E.ekari* group without lateral pronotal bead have rather strongly developed hook-like seta on male protarsomere 4, diminution of the hook-like seta was observed in some species of the *E.casuarina* group without the pronotal bead.

## ﻿Conclusion

New Guinean *Exocelina* represent a large and diverse group of Copelatinae beetles. Here, and in our previous publications (Table 1), we provide comprehensive taxonomic and faunistic treatments for this radiation. Further investigation of the group will definitely lead to more new species descriptions, some degree of restructuring of the species-group classification and better understanding of species distributions across the island. Having very diverse and intriguing character combinations, New Guinean *Exocelina* are an excellent potential model system for detailed studies on the evolution of homoplastic characters, co-evolution of different species, sexual dimorphism, and sexual conflict during mating.
